# Patients with primary carnitine deficiency treated with L‐carnitine are alive and doing well—A 10‐year follow‐up in the Faroe Islands

**DOI:** 10.1002/jmd2.12383

**Published:** 2023-09-11

**Authors:** Rannvá K. Abrahamsen, Allan M. Lund, Jan Rasmussen

**Affiliations:** ^1^ Gastro Unit, Medical Division Copenhagen University Hospital‐Amager and Hvidovre Hvidovre Denmark; ^2^ Department of Internal Medicine National Hospital Tórshavn Faroe Islands; ^3^ Department of Pediatrics and Clinical Genetics, Centre for Inherited Metabolic Diseases Copenhagen University Hospital, Rigshospitalet Copenhagen Denmark

**Keywords:** Faroe Islands, inherited metabolic disease, neonatal screening, primary carnitine deficiency, sudden death

## Abstract

Primary carnitine deficiency (PCD) can be lethal. Carnitine is essential for the transfer of long‐chain fatty acids across the inner mitochondrial membrane for β‐oxidation. The reported prevalence of PCD in the Faroe Islands of 1:300 is the highest in the world. The Faroese PCD patient cohort has been closely monitored and we now report results from a 10‐year follow‐up study of 139 PCD patients. Four patients have died of natural causes since diagnosis. There were no signs of cardiac complications related to PCD. 70.5% reported an effect of L‐carnitine treatment. 33.7% reported current symptoms with fatigue and low stamina being the most common. 65.1% had experienced side effects during L‐carnitine treatment. Most common side effects were fish odor, abdominal pain, and diarrhea. The overall mean L‐carnitine dosage was 66.3 mg/kg/day. Free p‐carnitine was similar between male and female patients on L‐carnitine—18.6 and 18.8 μmol/L, respectively. L‐carnitine supplementation seems to be a safe and effective treatment when suffering from PCD. PCD patients in the Faroe Islands are alive and doing well more than 10 years after diagnosis.

**Synopsis**

Patients with primary carnitine deficiency treated with L‐carnitine are alive and doing well more than 10 years after diagnosis.

## 1 INTRODUCTION

Primary carnitine deficiency (PCD) can be lethal.^1^ The sudden death of a young Faroese female in 2009, who had just been diagnosed with PCD, but had not yet received supplementary L‐carnitine treatment, sparked a public outcry for preventive measures. A voluntary screening program revealed a high prevalence of 1:300 in the Faroe Islands, a small island community in the North Atlantic with a population of only 54 000 inhabitants.^2^ In a postmortem study among Faroese individuals under the age of 45, who had died suddenly, a total of 13 were found with genetic analysis to have suffered from PCD.^1^ As a result, the Faroe Islands began to screen all newborns for PCD by measuring the carnitine level in their blood, 2–3 days after birth. Previous research has shown a direct correlation between the maternal and fetal carnitine levels.^3^ In order to accurately assess the infants carnitine level without any potential false elevation or reduction, a follow‐up blood test is offered 4 weeks after birth.

Carnitine is a quaternary amine and is essential for the transfer of long‐chain fatty acids across the inner mitochondrial membrane for β‐oxidation, and the export of acetyl‐ and chain‐shortened acyl products contributing to a stabile cell environment. Humans rely mostly on dietary sources for carnitine and an efficient renal reabsorption to maintain a proper level of carnitine in the body.^4^, ^5^, ^6^

PCD is an autosomal recessive disorder that affects the function of the organic cation transporter 2 (OCTN2) high‐affinity carnitine transporters, that localizes to the cell membrane and transport carnitine actively inside the cell. Without proper functioning OCTN2 carnitine transporters, the renal reabsorption of carnitine is impaired, and as a consequence, patients suffering from PCD have low plasma levels of carnitine. This can disturb cellular energy production and cause fatigue but also in extreme cases lead to cellular dysfunction and severe symptoms of coma and sudden death.^1^, ^7^, ^8^, ^9^, ^10^

Patients diagnosed with PCD in the Faroe Islands are treated with oral L‐carnitine supplementation. The screening efforts, which culminated in 2010, revealed a cohort of Faroese adults, which had not been diagnosed with the disorder prior to the screening program and thus had not received L‐carnitine supplementation. A study describing the health and cardiac status of 76 adult patients diagnosed with PCD in the Faroe Islands was published in 2014. It showed a marked improvement in self‐reported fatigue following the initiation of L‐carnitine treatment. But otherwise, most patients were asymptomatic.^11^

More than 10 years have now passed since the largest part of the PCD cohort was diagnosed with the disorder. Children diagnosed then have now become adults. Patients have been rigorously followed in an outpatient setting and the aim of this article is to report an ~10‐year follow‐up of the unique Faroese PCD patient cohort.

## 2 MATERIALS AND METHODS

All registered PCD patients born prior to 2014 and living in the Faroe Islands at the time of the follow‐up study were included. Furthermore, in patients who had emigrated from the Faroe Islands, we included data from their electronic health record, if they had been in contact with the Faroese health care system within 2 years of the study period from 2021 to 2022.

### 2.1 Questionnaire

All patients living in the Faroe Islands, above 18 and below 75 years of age and not suffering from other serious sickness, were contacted by phone if possible and invited to answer the following questions by the principle investigator: Symptoms before diagnosis. If they had experienced improvements in their symptoms following treatment. Current symptoms. If they had experienced side effects from the L‐carnitine treatment. Possible current side effects. Current dosage of L‐carnitine supplementation. Height and weight.

### 2.2 Blood tests

Hemoglobin, creatinine, Alanine Aminotransferase (ALAT) taken at least 5 years from the time of diagnosis. Mean plasma carnitine was calculated from the five most recently registered test results, though at least 5 years from diagnosis.

### 2.3 Echocardiography and electrocardiogram

Results from follow‐up echocardiography and electrocardiograms were only included if performed more than 5 years after diagnosis.

## 3 RESULTS

We included 139 PCD patients in the study—74 males and 65 females (Table 1). The mean age was 37.8 years with most patients between 30 and 45 years of age (*n* = 33). Height, weight, and body mass index for those above and below the age of 18 and 75, respectively were unremarkable and as expected in the Faroese population (Table 1). The overall mean L‐carnitine dosage was 66.3 mg/kg/day, with females tending to be on a higher dosage (68.7 mg/kg/day) than men (63.9 mg/kg/day; Table 1).

**TABLE 1:** Follow‐up characteristics, blood samples, echocardiography, and electrocardiogram.

		All subjects	Male	Female
		*n* = 139	*n* = 74	*n* = 65
<15 years		13	9	4
15–30 years		33	16	17
30–45 years		41	24	17
45–60 years		31	16	15
>60 years		17	7	10
Deceased		4	2	2
Mean age (years)		37.8 (18)	36.6 (17.5)	39.3 (18.7)
Height (cm)^a^		176 (9.8)	184 (6.8)	169 (6.0)
Weight (kg)^a^		83.9 (20.1)	95.1 (17.4)	73.5 (16.6)
Body mass index (kg/m^2^)^a^		26.9 (5.4)	28.2 (4.9)	25.8 (5.6)
L‐carnitine dosage (mg/kg)		66.3 (32.3)	63.9 (31.9)	68.7 (32.9)
Blood tests	Normal range			
Free p‐carnitine (μmol/L)^b^		18.7 (4.7)	18.6 (4.7)	18.8 (4.7)
Hemoglobin (mmol/L)		8.7 (0.8)	9.2 (0.7)	8.3 (0.5)
Creatinine (μmol/L)		72.9 (16.8)	80.5 (16.4)	65.2 (13.5)
ALAT (U/L)	10–70	30.7 (23.4)	36.7 (26.7)	25 (18.2)
Echocardiography^c^		*n* = 100		
Normal (%)		91		
Major changes (%)		2		
Minor changes (%)		7		
Electrocardiogram^c^		*n* = 100		
Normal (%)		83		
Major changes (%)		15		
Minor changes (%)		2		

*Note*: Mean (SD). Mean follow‐up time was 11.4 years (range 6–23 years).

^a^ Height, weight, and body mass index; *n* = 83, >18 years, and <75 years.

^b^ With L‐carnitine treatment.

^c^ Echo and ECG; *n* = 100, mean follow‐up time was 8.7 years (range 5–21 years).

Free p‐carnitine was similar between male and female patients on L‐carnitine—18.6 and 18.8 μmol/L respectively. Hemoglobin, creatinine, and ALAT were normal (Table 1).

Follow‐up echocardiography and electrocardiogram data were available in 100 patients with a mean follow‐up time of 8.7 years (Table 1). All echocardiography parameters were reported normal in 91%, while there were reported major and minor changes in 2% and 7%, respectively. The major changes were signs of mild to moderate left ventricular hypertrophy and a mildly reduced left ventricular ejection fraction (LVEF) of 45% in two middle‐aged male patients. Both had left bundle branch block. The minor changes ranged from mild valvular regurgitation to slight hypertrophy (Patient Table S1). Electrocardiograms were normal in 83% while there were signs of major and minor changes in 15% and 2% of the patients, respectively. Most major changes were atrial fibrillation and bundle branch block (Patient Table S1).

Four patients, two males and two females, have died since being diagnosed with PCD. They were 86, 81, 77, and 48 years of age and died of chronic obstructive pulmonary disease, ischemic cardiomyopathy and heart failure, lung cancer, and breast cancer.

Of the patients included in the questionnaire study (*n* = 83), 70.5% reported symptoms prior to treatment—and 70.5% reported an effect of L‐carnitine treatment (Table 2). A third of the patients (33.7%) reported current symptoms with fatigue and low stamina being the most common—especially females reported current symptoms (51.2%) compared with 15% in males (Table 2). Two thirds of the patients (65.1%) had experienced side effects during L‐carnitine treatment while a third (33.8%) was currently suffering from side effects (Table 2). Most common side effects were fish odor, abdominal pain, and diarrhea (Table 2).

**TABLE 2:** Questionnaire.

	Symptoms prior to treatment (%)	Effect of treatment (%)	Current symptoms (%)	Side effects during treatment (%)	Current Side effects (%)
Overall (*n* = 83)	70.5	70.5	33.7	65.1	33.8
Male (*n* = 40)	61.1	61.1	15	70	41.7
Female (*n* = 43)	78.6	78.6	51.2	60.5	32.5

Primary symptoms (%)^a^		Primary side effects (%)^b^	
Fatigue	43	Fish‐odor	35
Low stamina	22	Abdominal pain	23
Palpitations	7	Diarrhea	22
Muscle symptoms	7^c^		
Dizziness	5		
Difficulties gaining weight	5		

*Note*: Faroese PCD patients >18 and <75 years old living in the Faroe Islands.

Abbreviation: PCD, primary carnitine deficiency.

^a^ Other symptoms: Chest pain, exhaustion when hungry, frequent infections, constipation, craving meat, feeling cold, difficulty losing weight, poor sleep, and diarrhea.

^b^ Other side effects: Reflux, flatulens, sleeping sensation in limbs, hot flashes, leg cramps, nausea, headache, fatigue, and weight gain.

^c^ Muscle symptoms: Muscle pain, frequent sport injuries, and muscle cramps.

When stratified in relation to genotypes most patients were compound heterozygous for the c.95A > G (p.Asn32Ser) and the c.‐149G > A (*n* = 58), while 48 patients were homozygous for the c.95A > G mutation (Table 3). The c.95A > G homozygous group had a lower mean age than the other genotypes (27.6 years). And, the amount of daily L‐carnitine ingested was higher among c.95A > G homozygous patients (86.7 mg/kg/day) compared with the other genotypes.

**TABLE 3:** Genotype follow‐up characteristics, blood samples, mean (SD).

	c.95A > G	c.95A > G	c.95A > G	Other
	c.95A > G	c.‐149G > A‐5	c.825‐52G > A	
	*n* = 48	*n* = 58	*n* = 14	*n* = 19
Genotypes				
Male	27	22	13	12
Female	21	36	1	7
Mean age (years)	27.6 (17.1)	42.1 (15.7)	38.1 (11.8)	51 (18.6)
Dosage of L‐carnitine (mg/kg)	86.2 (35.8)	57.8 (23.4)	45.3 (12.2)	51.5 (30.2)
Blood tests				
Free p‐carnitine with treatment (μmol/L)	18.7 (5)	18.8 (4.6)	18.0 (4.7)	18.7 (4.5)
Free carnitine prior to treatment (μmol/L)^a^	2.0 (0.7)	3.5 (0.9)	4.2 (1.9)	N/A
Hemoglobin (mmol/L)	8.6 (0.9)	8.7 (0.7)	9.4 (0.7)	8.7 (0.6)
Creatinine (μmol/L/L)	70.2 (19.5)	71.5 (12.6)	86.1 (8.2)	77.4 (20.2)
ALAT (U/L)	23.1 (10.8)	33.3 (22.7)	52.9 (35)	21.2 (7.8)

	*n* = 20	*n* = 44	*n* = 10	*n* = 8
Follow‐up questionnaire				
Current symptoms (%)	35	32	0	37,5
Effect of L‐carnitine treatment (%)	87.5	65.9	66.7	75
Current side effects (%)	40	25	60	25

^a^ Reference: Residual OCTN2 transporter activity, carnitine levels, and symptoms correlate in patients with primary carnitine deficiency (Rasmussen et al. 2014; Table 1). c.95A > G (p.Asn32Ser).

The therapeutic reference was 15–25 μmol/L for all patients, and the free p‐carnitine during L‐carnitine treatment was kept in the similar range between the genotypes ranging from 18.0 to 18.8 μmol/L (Table 3).

There was no marked deviation between the genotypes regarding hemoglobin, creatinine, and ALAT (Table 3).

Almost 90% in the c.95A > G homozygous group reported a beneficial effect of L‐carnitine treatment, which also was the genotype with the lowest free carnitine prior to treatment (Table 3).

Forty‐eight patients had been admitted since being diagnosed (Patient Table S1). In the adult patients, the causes of admission to hospital do not seem to be related to PCD. In the children, it seems that they have been admitted quite often as a precautionary measure when experiencing signs of gastroenteritis and vomiting because their ability to adhere to the L‐carnitine treatment was affected.

## 4 DISCUSSION

The major finding in this study is, that almost all patients diagnosed with PCD more than 10 years ago and receiving L‐carnitine treatment are still alive and doing well, with the oldest female and male patients aged 89 and 81 years old, respectively. The four patients who have died since diagnosis died of natural causes. At least 13 sudden deaths among young Faroese individuals have been attributed to untreated PCD.^1^ PCD seems especially to have been a major cause of sudden unexplained death among young Faroese women, but the rigorous efforts to uncover undiagnosed PCD in the Faroe Islands might potentially prevent half of the sudden unexplained deaths in young Faroese females in the future.^1^

PCD patients seem to adhere well to L‐carnitine treatment, even though they have to ingest L‐carnitine tablets at least three times a day. The treatment ensures far higher levels of L‐carnitine in the blood compared with when not receiving L‐carnitine supplementation. This again leads to higher intracellular carnitine and stabilizes and protects against cellular dysfunction for example erratic cardiomyocytes and malignant cardiac arrhythmia.^12^

More than two‐thirds of the patients (70.5%) reported having had symptoms prior to initiation of treatment, while only a third (33.7) reported still suffering from especially fatigue and low stamina when on L‐carnitine. There can be little doubt, that L‐carnitine supplementation, as well as protecting against serious symptoms, also alleviates subtler symptoms of especially fatigue. But, it comes at a cost, because almost two‐thirds (65.1%) of the patients have experienced side effects. Although abdominal pain and diarrhea are a nuisance the more common side effect of bodily fish odor experienced by patients on L‐carnitine is potentially stigmatizing.^13^ Especially among the younger patients. Intestinal bacterial degradation of carnitine into trimethylamine (TMA), a nontoxic chemical with an unpleasant odor, is the cause of the unfortunate side effect. Advice given for reducing the odor includes frequent washing, use of antiperspirants, probiotic food supplements, and only rarely oral metronidazole.^13^ TMA is absorbed through the intestinal mucosa into the bloodstream and converted to trimethylamine‐N‐oxide (TMAO) in the liver.^14^ This leads to elevated levels of TMAO in patients supplemented with L‐carnitine.^15^ Increased levels of TMAO have been linked to an increased risk of atherosclerosis and cardiac disease.^16^, ^17^ We have not observed that Faroese PCD patients on high‐dose L‐carnitine supplementation have an increased risk of ischemic events—which also seems to be supported by previous reports.^15^, ^18^ We though need to investigate the Faroese PCD cohort more closely with regards to the association between elevated TMAO and atherosclerosis in order to rule it out.

There seems to be a gender difference in self‐reported current symptoms, as half (51.2%) of the females still report symptoms of especially fatigue compared with only 15% of the males—and females report less side effects (Table 2). Of the 13 reported sudden deaths in the Faroe Islands 10 were female.^1^ But, whether females really are at an increased physiological risk of adverse effects from PCD compared with males will be difficult to answer. We can at least see from our data, that the mean free p‐carnitine in both male and female PCD patients treated with L‐carnitine is almost identical (18.6 vs. 18.8 μmol/L).

Two males had mild left ventricular hypertrophy and mildly reduced LVEF. But both also had LBBB, which often causes a mild reduction in LVEF because of left ventricular dyssynchrony. They both suffered from hypertension and did not seem to benefit from L‐carnitine with regards to the mild hypertrophy, which likely is not related to PCD.

The PCD genotypes differ with regards to residual OCTN2 transporter activity and carnitine levels when untreated.^8^ Among the Faroese patients those homozygous for the c.95A > G mutation are especially vulnerable, as their residual OCTN2 transporter activity and free carnitine levels without treatment are the lowest among the different genotypes in the Faroe Islands.^8^ Furthermore, the 13 sudden death subjects were all homozygous for the c.95A > G mutation.^1^ Patients homozygous for the c.95A > G mutation receive more L‐carnitine supplement than the other genotypes in order to maintain p‐carnitine in the therapeutic range (Table 3). But, they also have the highest self‐reported benefit of L‐carnitine treatment (87.5%; Table 3).

Antibiotics containing pivalic acid have either been banned or are in strict limited use in the Faroe Islands since the discovery of a high prevalence of PCD. Pivalic acid is well known to cause an increased excretion of carnitine in urine.^19^, ^20^, ^21^ Several of the subjects who died suddenly of PCD had taken antibiotics containing pivalic acid shortly before their death.^9^ The far more cautious use of antibiotics containing pivalic acid in the Faroe Islands has and will continue to limit the risk of hazardous exposure to PCD patients—but only in the Faroe Islands. PCD patients traveling abroad are informed to be vigilant when being prescribed antibiotics in order to avoid being unnecessarily exposed to pivalic acid.

## 5 CONCLUSION

L‐carnitine supplementation seems to be a safe and effective treatment when suffering from PCD. PCD patients from the large Faroese PCD cohort treated with L‐carnitine are alive and doing well more than 10 years after diagnosis. A continued effort to diagnose PCD as early as possible in order to treat patients with L‐carnitine must be considered highly relevant.

## AUTHOR CONTRIBUTIONS

**Rannvá K. Abrahamsen:** Study design; collection of data; data analysis and preparation of article. **Jan Rasmussen:** Study design; data analysis and preparation of article. **Allan M. Lund:** Study design; preparation of article.

## FUNDING INFORMATION

National Hospital of the Faroe Islands (small local grant).

## CONFLICT OF INTEREST STATEMENT

Rannvá K. Abrahamsen, Jan Rasmussen, and Allan M. Lund declare that they have no conflict of interest.

## ETHICS STATEMENT

This study involved the analysis of anonymized data that had been previously collected. Additionally, the questionnaire was designed to ensure strict patient privacy and complete anonymity. Participants were fully informed about the purpose of the study and were given the voluntary choice to participate.

## INFORMED CONSENT

Informed consent was not required for this study as it involved the analysis of anonymized data that had been previously collected. Moreover, the questionnaire itself was designed to protect patient privacy by ensuring anonymity. Participants were fully informed about the purpose of the study and given the choice to participate.

## Supporting information


Table S1.

## Data Availability

The data supporting the findings of this study are available within the article and it's Patient Table S1. Raw data that support the findings of this study are available from the corresponding author, upon reasonable request.
