# Supplementary material for: Patients with primary carnitine deficiency treated with L‐carnitine are alive and doing well—A 10‐year follow‐up in the Faroe Islands
Source: JIMD Rep. 2023 Sep 11;64(6):453–9. doi: 10.1002/jmd2.12383 (PMC10623095; doi:10.1002/jmd2.12383)
Supplement: Supplementary file 1 — Table S1. [file JMD2-64-453-s001.pdf]

Ref. #	Sex	Age Years	*SLC22A5* mutations*** Allele 1	Allele 2	Follow-up Echo normal (yes/no) (M)=major (m)=minor	Follow-up ECG normal (yes/no) (M)=major (m)=minor	L-carnitine Dosage mg/kg	Symptoms before diagnosis*	Effect of L-carnitine*	Current symptoms*	Side effects of L-carnitine treatment*	Admissions since diagnosis	Cause of death
1	F	16	c.95A>G	c.695C>T	mild MR (m)	yes	62						
2	M	81**	c.95A>G	c.136C>G	N/A	LBBB (M)							COPD
3	M	26	c.95A>G	c.95A>G	yes	yes	182	diagnosed as infant	N/A	none	fish odour		
4	M	11	c.95A>G	c.95A>G	yes	yes	96						
5	M	20	c.95A>G	c.95A>G	yes	yes	64	diagnosed as infant	N/A	none	fish odour		
6	F	18	c.95A>G	c.95A>G	yes	yes	115						
7	M	14	c.95A>G	c.95A>G	yes	yes	99						
8	M	20	c.95A>G	c.95A>G	yes	yes	75	diagnosed as infant	N/A	none	fish odour		
9	F	45	c.95A>G	c.95A>G	yes	yes	92	low stamina	yes	none	fish odour, diarrhea		
10	M	36	c.95A>G	c.95A>G	N/A	N/A	90	none	no	none	fish odour, diarrhea		
11	F	19	c.95A>G	c.95A>G	yes	yes	79	diagnosed as infant	N/A	mild fatigue	fish odour, diarrhea		
12	F	15	c.95A>G	c.95A>G	yes	yes	64						
13	M	14	c.95A>G	c.95A>G	yes	yes	110						
14	M	37	c.95A>G	c.95A>G	yes	yes	82	fatigue	some	fatigue	diarrhea, abd. pain	suspected arrythmia	
15	F	15	c.95A>G	c.95A>G	yes	yes	208						
16	F	12	c.95A>G	c.95A>G	yes	yes	78						
17	F	12	c.95A>G	c.95A>G	yes	yes	73						
18	M	15	c.95A>G	c.95A>G	yes	yes	75						
19	M	81	c.95A>G	c.95A>G	mild PH (m)	AF, RBBB (M)	N/A					pulmonary embolism	
20	M	56	c.95A>G	c.95A>G	N/A	N/A	53					u.colitis	
21	F	41	c.95A>G	c.95A>G	N/A	N/A	72						
22	M	28	c.95A>G	c.95A>G	yes	yes	79	fatigue, low stamina	yes	none	fish odour, weight gain		
23	F	24	c.95A>G	c.95A>G	yes	yes	87	none	no	palpitations	diarrhea, abd. pain		
24	M	27	c.95A>G	c.95A>G	yes	ST depression (M)	55	low stamina	yes	none	none		
25	M	31	c.95A>G	c.95A>G	N/A	N/A	49	fatigue	yes	none	abd. pain, acid reflux	chest pain	
26	M	34	c.95A>G	c.95A>G	yes	neg. T-wave (M)	62					septic arthritis, SAH	
27	M	31	c.95A>G	c.95A>G	yes	yes	41	fatigue, low weight	yes	none	none		
28	M	41	c.95A>G	c.95A>G	yes	yes	46	none	no	none	diarrhea, abd. pain		
29	F	16	c.95A>G	c.95A>G	yes	yes	62					pneumonia, facial palsy	
30	F	25	c.95A>G	c.95A>G	yes	yes	56	fatigue, low stamina	yes	exhaustion	fish odour, abd. pain	syncope	
31	M	11	c.95A>G	c.95A>G	yes	yes	90					vomiting	
32	F	17	c.95A>G	c.95A>G	yes	yes	131					vomiting, UTI	
33	F	10	c.95A>G	c.95A>G	yes	yes	126					vomiting, respiratory infection	
34	M	86**	c.95A>G	c.95A>G	N/A	N/A	N/A						HF, infection
35	F	21	c.95A>G	c.95A>G	yes	yes	100						
36	M	22	c.95A>G	c.95A>G	yes	yes	82	fatigue, low stamina.	yes	none	abd. pain		
37	F	36	c.95A>G	c.95A>G	yes	N/A	100	fatigue	yes	mild fatigue	none	abdominal symptoms	
38	F	21	c.95A>G	c.95A>G	yes	yes	103	fatigue, low weight	yes	none	none	epileptic seizures	
39	M	15	c.95A>G	c.95A>G	yes	yes	104					vomiting, asthma attack, mono arthritis	
40	M	13	c.95A>G	c.95A>G	yes	yes	94					vomiting	
42	M	33	c.95A>G	c.95A>G	yes	yes	44	fatigue, low stamina.	yes	none	fish odour		
43	F	9	c.95A>G	c.95A>G	mild MR (m)	yes	159					vomiting	
44	F	33	c.95A>G	c.95A>G	yes	N/A	64	Fatigue, exhaustion	yes	exhaustion	abd. pain		
46	M	24	c.95A>G	c.95A>G	N/A	N/A	130						
48	M	47	c.95A>G	c.95A>G	N/A	N/A	49						
49	F	48	c.95A>G	c.95A>G	N/A	yes	51						
51	F	89	c.95A>G	c.95A>G	mild LVH (m)	AF, RBBB (M)	N/A					stroke, pneumonia, erysipelas	
52	M	9	c.95A>G	c.95A>G	N/A	N/A	N/A						
53	F	41	c.95A>G	c.95A>G	yes	yes	46	pale	some	fatigue	hot flashes, diarrhea	pulmonary embolism	
54	M	37	c.95A>G	c.95A>G	yes	yes	75	fatigue, low stamina.	yes	none	none		
55	M	20	c.95A>G	c.825-52G>A	yes	yes	61	diagnosed as infant	N/A	none	none	intra-abdominal abscess	
56	M	23	c.95A>G	c.825-52G>A	yes	yes	67						
57	M	45	c.95A>G	c.825-52G>A	yes	yes	38	none	some	fatigue, palpitations	diarrhea, abd. pain	syncope, appendicitis	
58	M	38	c.95A>G	c.825-52G>A	N/A	N/A	N/A						
59	M	44	c.95A>G	c.825-52G>A	yes	yes	33	none	no	none	none	chest pain	

60	M	18	c.95A>G	c.825-52G>A	yes	yes	51					pneumonia	
61	M	41	c.95A>G	c.825-52G>A	N/A	N/A	51	felt cold	yes	none	diarrhea, acid reflux		
62	M	45	c.95A>G	c.825-52G>A	mild LVH (m)	yes	31	none	no	none	fish odour		
63	M	51	c.95A>G	c.825-52G>A	LVH, LVEF 46% (M)	LBBB (M)	N/A						
64	F	56	c.95A>G	c.825-52G>A	N/A	N/A	52	muscle pain	yes	none	abd. pain, flatulens		
65	M	53	c.825-52G>A	c.825-52G>A	yes	yes	31	none	no	none	none		
66	M	42	c.825-52G>A	c.825-52G>A	yes	yes	35	difficulty building muscles	yes	none	fatigue		
67	M	47	c.95A>G	c.825-52G>A	N/A	N/A	42	felt cold	yes	none	acid reflux	cholecystectomy	
68	M	27	c.95A>G	c.825-52G>A	N/A	N/A	51	none	no	none	acid reflux		
69	M	15	c.136C>G	c.825-52G>A	yes	yes	142					abdominal symptoms	
70	M	35	c.95A>G	c.825-52G>A	N/A	N/A	40	fatigue	yes	none	fish odour		
71	M	44	c.95A>G	c.825-52G>A	N/A	N/A	27	infections	yes	none	diarrhea, abd. pain		
72	M	34	c.825-52G>A	c.-149G>A-5	N/A	N/A	64					spontaneous pneumothorax	
74	F	45	c.95A>G	c.-149G>A-5	yes	yes	78	none	no	none	fish odour		
75	M	38	c.95A>G	c.-149G>A-5	N/A	yes	98	none	no	none	fish odour, abd. pain	back pain	
77	M	53	c.95A>G	c.-149G>A-5	N/A	N/A	95	fatigue	yes	none	fish odour	myocardal infarction	
78	M	46	c.95A>G	c.-149G>A-5	N/A	N/A	32						
80	F	27	c.95A>G	c.-149G>A-5	N/A	N/A	N/A						
81	F	40	c.95A>G	c.-149G>A-5	yes	neg. T-wave (M)	67	fatigue, low stamina	yes	none	abd. pain	spontaneous pneumothorax	
82	F	55	c.95A>G	c.-149G>A-5	yes	ST depression (M)	52	fatigue	yes	none	fish odour		
83	F	28	c.95A>G	c.-149G>A-5	N/A	N/A	53						
84	M	27	c.-149G>A-5	c.-149G>A-5	yes	yes	31	none	no	fatigue	fatigue, low stamina		
85	F	65	c.95A>G	c.-149G>A-5	yes	yes	51	fatigue, muscle pain	some	fatigue	none		
86	M	52	c.95A>G	c.-149G>A-5	N/A	N/A	31	fatigue, sport injuries.	yes	palpitations	none		
87	F	69	c.95A>G	c.-149G>A-5	yes	yes	54	fatigue, infections	yes	none	weight gain, fish odour		
88	M	43	c.95A>G	c.-149G>A-5	yes	yes	37	fatigue, muscle cramps	yes	mild fatigue	muscle pain	mono arthritis	
89	F	70	c.95A>G	c.-149G>A-5	yes	RBBB, LAH (M)	42	low stamina	yes	none	fish odour	hysterectomy	
90	F	36	c.95A>G	c.-149G>A-5	yes	yes	27	fatigue, low stamina	yes	none	fish odour	cholecystectomy	
91	F	18	c.95A>G	c.-149G>A-5	yes	yes	58	none	no	none	fish odour		
92	M	58	c.95A>G	c.-149G>A-5	yes	N/A	N/A					chest pain, cholecystectomy	
94	F	38	c.95A>G	c.-149G>A-5	yes	yes	39	fatigue, infections	yes	fatigue, palpations	fish odour, diarrhea	myocardial infarction, chest pain	
95	F	58	c.-149G>A-5	c.-149G>A-5	N/A	N/A	57	fatigue	yes	mild fatigue	fish odour, abd. pain	diverticulitis	
96	M	43	c.95A>G	c.-149G>A-5	yes	yes	38	none	no	none	fish odour		
97	M	30	c.95A>G	c.-149G>A-5	yes	yes	40					seizure	
98	F	30	c.95A>G	c.-149G>A-5	yes	1st AVB (m)	83	fatigue, muscle cramps	yes	mild fatigue	none		
101	M	60	c.95A>G	c.-149G>A-5	yes	yes	29	none	no	none	none	carotic dissection	
102	F	53	c.95A>G	c.-149G>A-5	yes	yes	125	fatigue, chest pain	yes	none	fish odour		
103	F	37	c.95A>G	c.-149G>A-5	yes	yes	66	fatigue, poor appetite	yes	chest pain	none		
104	F	18	c.95A>G	c.-149G>A-5	yes	N/A	61						
105	F	33	c.95A>G	c.-149G>A-5	yes	yes	67	low stamina	yes	none	none		
106	M	18	c.95A>G	c.-149G>A-5	yes	yes	82	low stamina	yes	none	fish odour, diarrhea	vomiting	
107	F	48	c.95A>G	c.-149G>A-5	N/A	N/A	58	none	no	none	none		
108	M	37	c.95A>G	c.-149G>A-5	yes	N/A	50	chest pain	yes	none	none	chest pain	
109	M	56	c.-149G>A-5	c.-149G>A-5	N/A	N/A	N/A						
110	M	77	c.-149G>A-5	c.-149G>A-5	LVH, LVEF 45% (M)	AF, LBBB (M)	20					AF, herniotomy	
112	F	29	c.95A>G	c.-149G>A-5	N/A	yes	N/A	fatigue, frequent fainting	yes	palpitations	fish odour, abd. pain	chest pain, syncope, haematemesis	
113	M	30	c.95A>G	c.-149G>A-5	yes	yes	46	none	no	none	fish odour		
115	F	40	c.95A>G	c.-149G>A-5	yes	yes	56	none	no	chest pain	none		
116	F	65	c.95A>G	c.-149G>A-5	yes	N/A	69	fatigue	yes	mild fatigue	none		
117	F	31	c.95A>G	c.-149G>A-5	yes	yes	66	fatigue, low weight	yes	fatigue, chest pain	fish odour, acid reflux		
118	F	61	c.-149G>A-5	c.-149G>A-5	N/A	N/A	N/A						
119	M	54	c.-149G>A-5	c.-149G>A-5	yes	yes	41	fatigue	some	none	none	UTI	
120	F	77**	c.95A>G	c.-149G>A-5	AR, mild LVH (m)	N/A	N/A						lung cancer
121	F	51	c.95A>G	c.-149G>A-5	yes	N/A	54						
122	F	51	c.95A>G	c.-149G>A-5	yes	yes	33	palpitations, constipation	some	palpitations	fish odour	supraventriclar tachycardia	
123	M	43	c.95A>G	c.-149G>A-5	yes	N/A	60	dizzyness	yes	none	none	back pain	
124	M	67	c.-149G>A-5	c.-149G>A-5	N/A	N/A	N/A						
125	M	8	c.95A>G	c.-149G>A-5	yes	N/A	84					fever	
128	M	46	c.95A>G	c.-149G>A-5	N/A	N/A	33	none	no	none	diarrhea		
129	F	71	c.-149G>A-5	c.-149G>A-5	N/A	N/A	38	mild fatigue	yes	none	none		
130	F	48**	c.95A>G	c.-149G>A-5	N/A	N/A	N/A						breast cancer
131	F	42	c.95A>G	c.-149G>A-5	yes	yes	111	none	some	none	none		

132	M	48	c.95A>G	c.-149G>A-5	yes	yes	35	low stamina	some	none	headache		
133	F	53	c.95A>G	c.-149G>A-5	N/A	N/A	53	none	no	none	none		
134	F	22	c.95A>G	c.-149G>A-5	yes	yes	57	fatigue	some	fatigue	none		
135	M	75	c.-149G>A-5	c.-149G>A-5	yes	AF (M)	N/A					AF, surgery renal tumor, PCI	
136	F	30	c.95A>G	c.-149G>A-5	N/A	N/A	56	none	some	none	fatigue, weight gain		
138	M	38	c.95A>G	c.-149G>A-5	yes	yes	99	low stamina	yes	none	none		
139	F	40	c.95A>G	c.-149G>A-5	yes	yes	26	chest pain	yes	fatigue	fish odour		
140	F	59	c.95A>G	c.-149G>A-5	yes	yes	56	exhausted when hungry	no	exhaustion	fish odour		
142	F	64	c.-149G>A-5	c.-149G>A-5	yes	N/A	54	mild fatigue	yes	none	none	u.colitis, pneumonia	
143	F	48	c.-149G>A-5	c.-149G>A-5	yes	yes	47						
145	M	31	c.95A>G	c.-149G>A-5	N/A	N/A	N/A						
146	F	53	c.95A>G	c.-149G>A-5	yes	Bradycardia (m)	25	fatigue	no	fatigue	fish odour		
147	M	63	c.95A>G	c.-149G>A-5	N/A	N/A	N/A						
148	M	57	c.-149G>A-5	c.-149G>A-5	N/A	AF (M)	N/A						
149	F	46	c.95A>G	c.-149G>A-5	yes	yes	43	fatigue, headache	yes	headache	abd. pain, fish odour		
150	M	14	c.95A>G	c.-149G>A-5	yes	yes	87						
151	F	66	c.95A>G	c.-149G>A-5	yes	LBBB (M)	56	none	no	none	none	pancreatitis	
152	F	43	c.-149G>A-5	c.-149G>A-5	yes	yes	48	Fatigue, exhaustion	some	exhaustion	nausea	stroke	
153	M	62	c.95A>G	c.-149G>A-5	MR (m)	yes	43	fatigue, muscle pain	no	fatigue,muscle pain	none	chest pain, periappendicular abscess	
154	F	69	c.95A>G	c.-149G>A-5	yes	LBBB (M)	49	none	no	none	none		
155	F	40	c.95A>G	c.-149G>A-5	N/A	N/A	38	low stamina	yes	none	none		
157	M	6	c.95A>G	c.-149G>A-5	N/A	N/A	100						

*Responded to questionary. **Deceased. *** c.95A>G (p.Asn32Ser); c.136C>T (p.Pro46Ser); c.131C>T (p.Ala44Val); c.695C>T (p.Thr232Met); c.825-52G>A (Unknown); c.-149G>A-5 (5 prime UTR variant)
